# Evaluation of Different Polysaccharide–Iron Complex Preparations In Vitro and In Vivo

**DOI:** 10.3390/pharmaceutics17030292

**Published:** 2025-02-23

**Authors:** Xin Yan, Qi Zhang, Tao Wang, Yu Luo, Xianyi Sha

**Affiliations:** School of Pharmacy, Fudan University, Shanghai 201203, China; 24111030090@m.fudan.edu.cn (X.Y.); zhangqi22@m.fudan.edu.cn (Q.Z.); 21211030097@m.fudan.edu.cn (T.W.); 20301030040@fudan.edu.cn (Y.L.)

**Keywords:** iron-deficiency anemia, polysaccharide–iron complex, iron supplement preparation, pharmacokinetics, bioavailability

## Abstract

**Objectives**: Iron-deficiency anemia is one of the most common nutritional deficiencies worldwide. Polysaccharide–iron complexes (PICs), as novel organic iron supplements, have garnered increasing attention due to their high bioavailability, minimal gastrointestinal irritation, and favorable tolerability. However, different formulations of PICs can show significant variations in their physicochemical properties and bioavailability. These factors are crucial for clinical efficacy and safety. **Methods**: This study selected two formulations of polysaccharide–iron complexes: Formulation A (PIC-coated pellets) and Formulation B (PIC powders), with ferrous succinate tablets (Formulation C) used as a control. The focus was on evaluating the molecular weight of the polysaccharides, the levels of free iron, and the dissolution across various dissolution media. Physicochemical properties were compared through particle size analysis, dissolution rate testing, and free iron content determination. Additionally, the pharmacokinetic properties of the two PIC formulations were assessed in a beagle dog model of iron-deficiency anemia. **Results**: Significant differences were observed in particle appearance and content structure between the two PIC formulations. Formulation A, prepared using pellet technology, exhibited a uniform particle size distribution. Its dissolution rate in acidic environments was significantly lower than that of Formulation B. In simulated gastric fluid, the cumulative iron dissolution rate of Formulation A was less than 15% within two hours, while that of Formulation B exceeded 50%, with substantial batch-to-batch variability. In various dissolution media, Formulation A released 12% of its dissolved iron content in gastric fluid within two hours. In contrast, the absolute free iron content of Formulation B was 8.5 times higher than that of Formulation A in simulated gastric fluid. In the beagle dog model of iron-deficiency anemia, Formulation A showed significantly higher bioavailability, which suggests that the pellet preparation technology improves both the acid resistance and bioavailability of the PIC formulation. **Conclusions**: The study revealed that Formulation A, prepared using pellet technology, possesses unique quality characteristics. This technology significantly reduces the release of free iron from PICs due to gastric acid action, potentially minimizing gastrointestinal irritation. Moreover, the pellet preparation process improves the acid resistance and bioavailability of PIC formulations, offering a more effective therapeutic option for iron-deficiency anemia. Future research may further explore the potential applications of pellet technology in other iron supplement formulations.

## 1. Introduction

Iron is essential for all living organisms and is one of the most abundant trace elements. Within the human body, iron can be classified by various criteria, with a common classification distinguishing between functional iron and non-functional storage iron based on its role. Functional iron makes up about 70% of total body iron and is mainly found in hemoglobin, myoglobin, and other oxygen-carrying proteins, as well as heme enzymes and certain cofactors that play a critical role in the respiratory chain. Non-functional storage iron, predominantly existing as ferritin and hemosiderin, is mainly stored in the spleen, liver, and bone marrow, serving as the body’s iron reservoir [1]. Iron is pivotal in the respiratory chain, and its deficiency can lead to various metabolic disturbances, such as impaired oxygen transport and utilization, thereby adversely affecting growth, development, and even causing neuropsychiatric abnormalities and anemia [2,3,4]. Anemia is a spectrum of disorders, including chronic kidney disease anemia, pregnancy-related anemia, and tumor-associated anemia. Iron-deficiency anemia (IDA) [5,6] results from low iron levels and affects more than 25% of the global population. It accounts for over 50% of all anemia cases [7]. The incidence of IDA is particularly high among pregnant women and children, primarily due to their increased nutritional needs for growth and development [8].

Treatment for iron-deficiency anemia (IDA) should be customized based on the severity of the condition and its cause. Blood transfusions may be required in severe or acute cases of anemia. Intravenous iron supplementation, a novel approach, involves the direct infusion of iron isomaltose or polysaccharide–iron complexes into the bloodstream. This method allows macrophages to absorb and gradually release iron, replenishing the levels of circulating iron. In clinical practice, moderate or mild IDA cases are often managed with oral iron supplements, including ferrous sulfate, ferrous succinate, ferrous fumarate, ferrous gluconate, and ferrous glycine. Traditional Chinese medicine approaches may also involve dietary strategies using iron-rich foods, such as fish, poultry, organ meats, and blood products, alongside supplements like animal-hide gelatin, which can enhance hematopoietic and immune functions. A wide variety of iron supplementation preparations are currently available, with amino acid iron chelates increasingly attracting the attention of researchers. Recent studies have focused on inorganic iron, organic acid iron, and emerging iron preparations. The first-generation iron supplements are traditional inorganic ferrous salts, such as ferrous sulfate, while the second-generation iron supplements are low-molecular-weight organic acid salts represented by ferrous fumarate and ferrous succinate, in which iron exists in the form of ferrous ions. These chemically unstable ferrous salts are prone to generating free radicals, leading to gastrointestinal side effects, such as nausea, vomiting, diarrhea, and constipation. They can also cause oxidative damage to cell membranes [9]. Moreover, studies have shown that the use of iron salts may affect the composition and function of the gut microbiota [10]. Therefore, exploring and developing novel iron carrier systems with high bioavailability and better stability is of significant importance. Polysaccharide–iron complexes (PICs), as third-generation oral iron supplements, have garnered significant interest due to their excellent solubility and high in vivo bioavailability [11,12,13]. PICs are primarily produced through chemical synthesis, which relies on stable complexation reactions instead of mere physical adsorption [14]. The mechanisms of binding between polysaccharides and iron include the site-binding model [15] and the colloidal model. For example, the binding of dextran with iron conforms to the colloidal model, where iron(III) ions polymerize to form a β-FeOOH iron core through oxygen and hydroxyl bridges. The terminal carboxyl groups of the polysaccharide envelop the iron core surface via covalent bonds to form the complex. Under neutral pH conditions, intrinsically anionic carbohydrates (such as glucuronates) or partially oxidized polysaccharides serve as more effective stabilizers. In this context, carboxyl groups provide negative charges over a broad pH range, and their chelation with iron can further promote the deprotonation of adjacent hydroxyl groups, thereby enhancing the stability of the complex [16,17,18]. Currently, various drug delivery systems and preparation techniques can be employed to optimize the properties of pharmaceuticals, such as liposomes, microspheres, and hydrogels, to prolong drug retention in the body and enhance bioavailability. Microspheres are multi-unit oral dosage forms that can release iron in the specific environment of the gastrointestinal tract. Each dose can consist of tens to hundreds of spherical pellets, which can be either encapsulated or compressed into tablets. They are primarily administered orally, exhibit good flowability, and have relatively stable drug release rates. Pelletization can prevent iron from contacting the external environment, delay its release in the gastric environment, and prolong its retention in the intestines. Liposomes are closed vesicles composed of a bilayer structure made of phospholipids and cholesterol [19]. Encapsulating iron within liposomes can improve its absorption, tolerability, and bioavailability. Structural changes in circulating liposomes [20] can be triggered by external stimuli, such as pH changes, ionic concentrations, and temperature gradients, which can release the embedded iron. Compared with traditional iron supplements, iron encapsulated in liposomes can achieve significantly enhanced absorption efficiency.

The present study was designed to systematically investigate the critical quality attributes of the original polysaccharide–iron complex capsule (hereinafter referred to as test sample A), which was the first polysaccharide–iron complex capsule launched in China in 1996. The investigation encompassed the polysaccharide molecular weight, free iron content, dissolution characteristics in various media, and pharmacokinetic properties in vivo. Comparative analyses were performed on different iron preparations, with a focus on molecular weights, free iron levels, and dissolution profiles. The overarching goal of this study was to establish essential technical quality parameters that reflect the unique strengths and characteristics of the original drug, thereby providing a reference for rational drug selection in clinical practice.

## 2. Materials and Methods

### 2.1. Materials

The following materials were used in the study: NaOH, HCL, KH_2_PO_4_, C_12_H_25_NaSO_4_, CH_3_COONa, C_2_H_4_O_2_, trypsin, and pepsin for the first type of polysaccharide–iron complex capsule (Formulation A, code: test sample A, imported coated pellets marketed Niferex capsules, 150 mg, produced by USA, Indiana, Kremers Urban Pharmaceuticals); the active ingredient was the iron element in the form of a polysaccharide–iron complex molecule, with each capsule containing 0.15 g of iron. The excipients included a sucrose pellet core, medicated glaze, povidone, and hydroxylated castor oil. For the second type of polysaccharide–iron complex capsule (Formulation B, code: test sample B, locally marketed powders in capsules, 150 mg), the active ingredient was the iron element in the form of polysaccharide–iron complex molecules, which appeared as a brown or brown-black crystalline powder. Each capsule contained 0.15 g of iron. The ferrous succinate tablets (Formulation C, code: test sample C, locally marketed film-coated tablets, 100 mg) contained ferrous succinate at 0.1 g, with excipients including succinic acid, calcium hydrogen phosphate, corn starch, polyvinyl ketone K30, magnesium stearate, and a film-coated premix. Polysaccharide–iron complexes (PICs, 46%, powder) are the active ingredients in the imported Niferex capsules.

### 2.2. Morphology and Physical Properties

#### 2.2.1. Morphology

The surface morphology of the samples was characterized using scanning electron microscopy (SEM). The sample preparation procedure was as follows: Initially, conductive tape was affixed to the sample holder, followed by uniform deposition of the sample onto its surface. Any loosely adhered particles were subsequently removed using an air puffer, ensuring optimal sample presentation for electron microscope observation. In instances where the samples exhibited inadequate electrical conductivity, a conductive film coating was applied prior to electron microscope examination.

#### 2.2.2. Molecular Weight and Distribution

##### Instruments, Reagents, and Chromatographic Conditions

Instruments: Agilent HPLC/RID detection system; electronic balance (Göttingen, Germany, CP225D sartorius company).

Reagents: A series of polysaccharide molecular weight control products (Shodex standard P-82, Lot No.: 130901); polysaccharide–iron complex capsules (test sample A); polysaccharide–iron complex capsules (test sample B). NaH_2_PO4, Na_2_HPO_4_, NaCl, and NaN_3_ were all domestic analytically pure. The water was ultra-pure.

Chromatographic conditions: Agilent gel column in series; mobile phase: pH 6.8 phosphate buffer (NaH_2_PO_4_ and Na_2_HPO_4_, 25 mmol/L; NaCl, 50 mmol/L; 0.02% sodium azido). Flow rate: 0.5 mL/min; sample size: 100 μL; column temperature: room temperature (25 °C).

##### Test Methods

Preparation of reference solution: Six polysaccharide reference products with molecular weights of 6200, 10,000, 21,700, 48,800, 113,000, and 200,000 were added to dissolve and dilute in a solution of 5 mg/mL (overnight).

Preparation of polysaccharide–iron complex (test sample A) test solution: Accurately weigh the products in quantities of 0.50 g and 0.25 g, place them in a 50 mL bottle, add the appropriate amount of mobile phase, ultrasonicate them for 20 min, cool them, dilute them with mobile phase to scale, shake well, leave them at room temperature overnight, filter, take further filtrate, and obtain.

Preparation of polysaccharide–iron complex (test sample B) test solution: Accurately weigh the products in quantities of 0.50 g and 0.25 g, respectively; place them in a 50 mL bottle; add the appropriate amount of mobile phase; ultrasonicate them for 20 min; cool them; dilute them with mobile phase to scale; shake well; leave them overnight at room temperature; filter; take further filtrate; and obtain.

Test method: Take 100 μL quantities of the control solution and the test solution; inject them into the liquid chromatograph; and determine them according to the chromatographic conditions described in the section on Instruments, Reagents, and Chromatographic Conditions.

### 2.3. Determination of Dissolution of Different Polysaccharide–Iron Complex Preparations

#### 2.3.1. Instruments and Reagents

The following instruments and reagents were used in this study: an ICPOES 5900 with an SPS4 automatic sampler (Agilent Corp, Santa Clara, CA, USA,), ultra-pure water (Merker-Millipore ultra-pure water meter), concentrated nitric acid (ampere CNW), and iron standard solution (5190-8285).

#### 2.3.2. Preparation of Four Dissolution Media

##### Artificial Gastric Fluid (AGF)

Take 16.4 mL diluted hydrochloric acid, add 800 mL water and 10 g pepsin with water, and dilute to 1000 mL, in which dilute hydrochloric acid is 234 mL concentrated hydrochloric acid, and dilute to 1000 mL with water, to obtain 9.5–10.5% dilute hydrochloric acid.

##### Acetic Acid Buffer (1% Sodium Dodecyl Sulfate)

Take 18 g CH_3_COONa, 9.8 mL C_2_H_4_O_2_, and 10g C_12_H_25_NaSO_4_ and add them to 1000 mL of water and stir to dissolve.

##### Artificial Intestinal Fluid (AIF)

Dissolve 6.8 g of KH_2_PO_4_ with 500 mL of water and adjust the pH to 6.8 with 0.4% (*w*/*w*) NaOH. Dissolve 10 g trypsin with the appropriate amount of water, mix the two liquids, add water to 1000 mL, and mix well.

##### pH 7.5 Buffer Solution (1% Sodium Dodecyl Sulfate)

Add 1000 mL water to 6.33 g KH_2_PO_4_ and 1.52 g NaOH to dissolve them. If necessary, add phosphoric acid solution or sodium hydroxide solution to adjust the pH value to 7.50 ± 0.05. Add 10 g C_12_H_25_NaSO_4_ and stir to dissolve.

#### 2.3.3. Measurement Methods

Take one prepared sample or accurately weigh 326 mg of PIC powder (equivalent to 150 mg of iron) and place it into the settling basket (directly adding the PIC powder into the disintegrating cup). Following the dissolution and release method outlined in the 2020 edition of the *Chinese Pharmacopoeia*, General Rule 0931 (the second method), utilize a disintegrating medium volume of 900 mL at a speed of 100 revolutions per minute. According to the protocol, withdraw 10 mL samples at various time intervals, filter them, and utilize the resultant filtrate as the test solution. Simultaneously, add 10 mL of blank dissolution medium.

Additional appropriate volumes of iron standard solution were measured and individually prepared in a series of standard curve solutions at concentrations of 0.5, 1, 5, 10, 50, 100, and 200 μg/mL using a 2% dilution of nitric acid. These solutions served as control samples. Following this, suitable volumes of both the test solution and control solutions were employed, and the iron content was quantified utilizing the ICP method.

#### 2.3.4. Determination of Free Iron Content During Dissolution of Different Polysaccharide–Iron Complex Preparations

The samples were obtained from the dissolved samples of each preparation in various dissolution media over a 2 h period. After sampling for 120 min, 50 mL of liquid was promptly withdrawn from the dissolving cup and filtered using a 0.45 μm filter membrane. Subsequently, 10 mL of the filtrate was transferred into a 15 mL centrifuge tube and labeled as sample 1. An additional 9 mL of the liquid was introduced into a 3 kDa PES ultrafiltration tube and centrifuged at 4000 g for 60 min. The supernatant was carefully collected from the outer tube and transferred into a 15 mL centrifuge tube for storage. The filtrate obtained from the ultrafiltration process constituted the sample for measuring free iron. The concentration of free iron was determined using ICP analysis, enabling the calculation of the percentage of free iron in the total iron content.

### 2.4. In Vivo Pharmacokinetic Studies

#### 2.4.1. Animals

Eight healthy female beagle dogs, approximately 10 months old and weighing approximately 8 kg, were purchased from Shanghai Jiao Tong University Agricultural Student Experimental Internship Field Co., Ltd. (Shanghai, China). All the animal experimental procedures were in accordance with the guidelines for the Care and Use of Laboratory Animals of Fudan University and approved by the Institutional Animal Care and Use Committee of the School of Pharmacy, Fudan University.

#### 2.4.2. Induction of Iron-Deficiency Anemia (IDA)

An IDA model in adult female beagle dogs was established by combining an iron-deficient diet (IDD) with regular phlebotomy. The success of the model was determined based on hemoglobin (Hgb) concentration thresholds. Throughout the model induction and experimental period, the dogs were fed exclusively with an IDD and ultra-pure water until the completion of the study. Prior to the onset of the experiment, the dogs were gradually transitioned to the IDD. This was achieved by mixing the IDD (iron content: 15 ppm) with normal feed and progressively increasing the proportion of the IDD. By day 14, the normal feed was completely replaced with the IDD, and ultra-pure water was provided as the sole drinking water source. Following the dietary transition, the IDD served as the only nutritional source for the beagle dogs. To induce anemia, phlebotomy was performed twice a week, with 20 mL of blood withdrawn each time, for a total duration of 8 weeks. Complete blood counts (CBCs) were measured every 14 days. The IDA model was considered successfully established when the Hgb concentration decreased to 80% of the baseline value (day 0).

#### 2.4.3. Pharmacokinetic Study Design

Prior to the experiments, eight IDA female beagle dogs (10–12 kg) were fasted for 8 h and divided into two groups (*n* = 4 per group): Group 1 received the first type of polysaccharide–iron complex capsule (test sample A, Niferex capsule, 150 mg), and Group 2 received the second type of polysaccharide–iron complex capsule (test sample B, powders in capsule, 150 mg). The pharmacokinetic profiles of test sample A and test sample B in the dogs were evaluated in a randomized, single dose, 2 × 2 crossover study with a 2-week washout period. After the IDA dogs were orally administered test sample A (one capsule) or test sample B (one capsule), blood samples were collected at 0.5, 1,1.5, 2, 2.5, 3, 3.5, 4, 5, 6, 8, 12, and 24 h. Blood samples were centrifuged at 12,000 rpm for 10 min, and the obtained plasma samples were kept frozen at −80 °C until analyzed by inductively coupled plasma mass spectrometry (ICP MS7900, California, USA, Agilent Corp).

### 2.5. Statistical Analysis

Data are expressed as means and standard deviations (SDs). Treatment means were compared by one-way ANOVA, and pairwise comparisons were made by the least significant difference (LSD) test. Differences were considered statistically significant when *p* ≤ 0.05. The area under the curve (AUC) from time zero until the last measured concentration (AUC_0–t_) was calculated by the trapezoidal method. The AUC from time zero to infinity (AUC_0–∞_) was obtained by adding the AUC from time zero to the last measured concentration (AUC_0–t_) to the extrapolated area, which was calculated as the last measured plasma concentration divided by the elimination rate constant. C_max_ was calculated as the measured maximum plasma concentration. T_max_ was calculated as the time to reach C_max_.

## 3. Results

### 3.1. Appearance and Apparent Morphology

**Figure 1:**
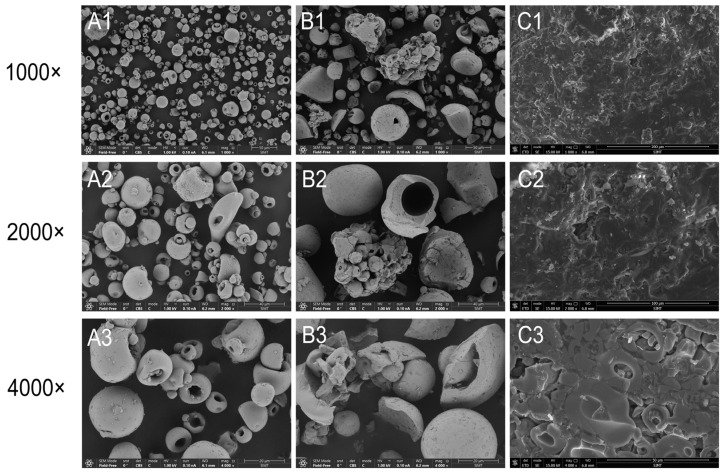
SEM results of polysaccharide–iron complexes ((**A1**–**A3**): PIC powders; (**B1**–**B3**): test sample B, powders in capsule, 150 mg; (**C1**–**C2**): inner coating of test sample A; (**C3**): outer coating of test sample A).

**Figure 2:**
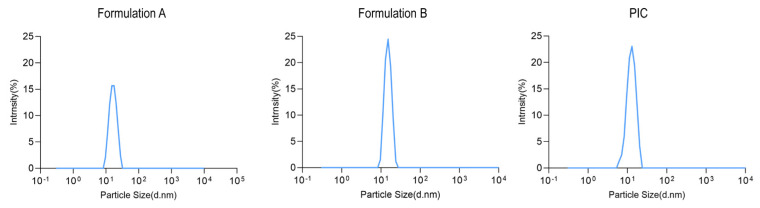
The particle size distribution diagrams of PICs in Formulation A, PICs in Formulation B, and the raw material (PICs).

The contents of test sample A consisted of brown pellet particles, while test sample B contained brown powder, similar to the PIC sample, which was also in powdered form. Moreover, the morphology of the samples was analyzed using scanning electron microscopy (SEM). The SEM results revealed that the PIC powder from the raw material comprised irregular spheres ranging from 3 to 40 μm, with a predominant size range of approximately 20 μm. These microspheres exhibited a distinct concave structure resembling a teacup (Figure 1(A1–A3)). Similarly, test sample B exhibited irregular spheres ranging from 10 to 50 μm, also displaying the characteristic hollow teacup shape. However, compared to the PIC powder, test sample B featured larger particle sizes, a non-uniform particle size distribution, and significant shape discrepancies among particles, including the presence of large shell fragments and small spheroid aggregates (Figure 1(B1–B3)), possibly attributable to excipient particles within the preparation. Test sample A appeared as spheres with a diameter of approximately 1 mm, featuring an inner layer with a diameter of about 0.5 mm composed of a sucrose pellet core surrounded by a layer of polysaccharide–iron complexes and excipients. The inner profile of test sample A exhibited a dense and irregular crystalline structure (Figure 1(C1,C2)), while the outer profile displayed a layered structure formed by the aggregation of hollow spheres (Figure 1(C3)). The morphology of these hollow spheres closely resembled that of the bulk drug powder, and the characterization results are depicted in Figure 1. The particle size distribution results of different iron preparations after dissolution in pH 6.8 buffer are shown in Figure 2. The particle size measurements following dissolution in pH 6.8 buffer solution revealed that the average particle size of the PICs in Formulation A was 14.45 ± 3.65 nm, with a polydispersity index of 0.2. For the PICs in Formulation B, the average particle size was 16.95 ± 4.82 nm, and the polydispersity index was 0.18. The raw material (PICs) exhibited an average particle size of 14.58 ± 3.22 nm, with a polydispersity index of 0.23.

### 3.2. Molecular Weight and Distribution

Table 1 displays notable differences in the weight-average molecular weight (Mw) and number-average molecular weight (Mn) between the two polysaccharide–iron complex preparations, with test sample A exhibiting a significantly higher relative molecular weight compared to test sample B. Moreover, the relative molecular weight of the polysaccharide–iron complexes in test sample A corresponds to that of the raw PICs. Additionally, the molecular weights of the various polysaccharide–iron complexes exhibit wide distribution characteristics (polydispersity index, PDI > 1.5).

### 3.3. Dissolution of Different Polysaccharide–Iron Complex Preparations in Various Media

#### 3.3.1. Dissolution of Different Polysaccharide–Iron Complex Preparations in Artificial Gastric Fluid

**Figure 3:**
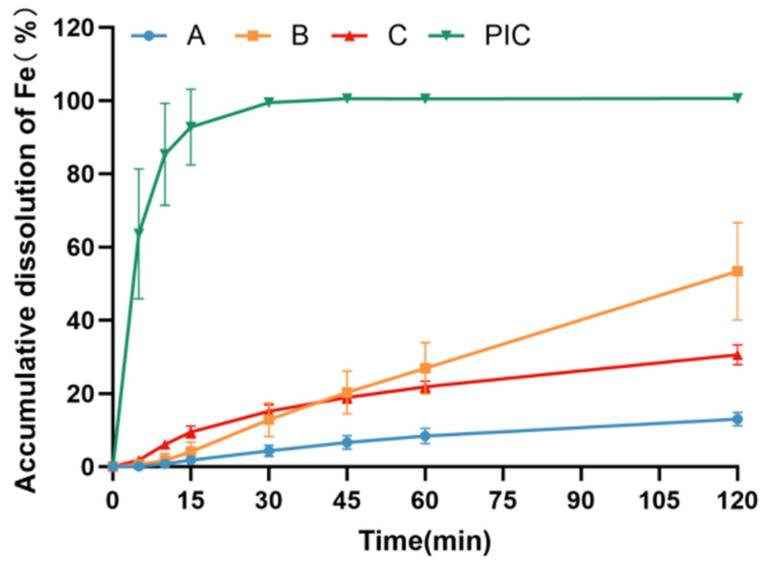
Dissolution curves of different iron preparations in artificial gastric fluid. Test sample A: coated pellets in imported Niferex capsules, 150 mg; test sample B, powders in capsule, 150 mg; test sample C: ferrous succinate tablet, film coating tablets, 100 mg; PIC: powders, the active ingredients in imported Niferex capsules.

The findings depicted in Figure 3 reveal substantial disparities in the dissolution behavior of various iron preparation samples in artificial gastric fluid. Notably, the dissolution rate of test sample A is relatively sluggish, with the cumulative dissolution rate at 2 h being less than 15%. Conversely, the dissolution rate of the polysaccharide–iron complex resembling test sample B is comparatively rapid, achieving an iron dissolution rate exceeding 50% within 2 h. This discrepancy could be attributed to the favorable solubility of PICs in artificial gastric fluid, facilitating their rapid dissolution.

#### 3.3.2. Dissolution of Different Polysaccharide–Iron Complex Preparations in pH 4.5 Buffer Solution

**Figure 4:**
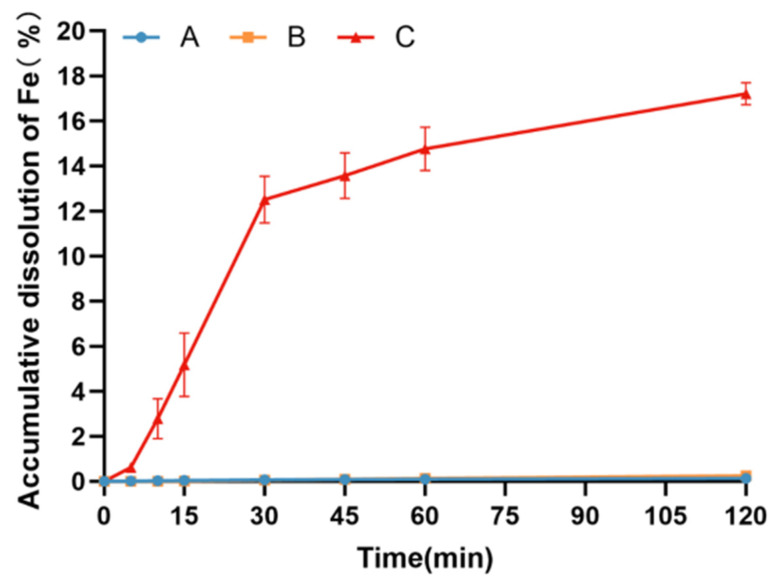
Dissolution curves of different iron preparations in pH 4.5 buffer. Test sample A: coated pellets in imported Niferex capsules, 150 mg; test sample B, powders in capsules, 150 mg; test sample C: ferrous succinate tablets, film coating tablets, 100 mg.

The outcomes depicted in Figure 4 highlight substantial variations in the dissolution behavior of diverse iron preparation samples in pH 4.5 buffer. Particularly noteworthy is the significantly higher dissolution rate observed in organic iron preparations compared to polysaccharide–iron complex preparations (*p* < 0.01). Notably, the dissolution rates of both polysaccharide–iron complex preparations were notably slow, with the cumulative dissolution rate at 2 h being less than 1%. This observation is likely attributable to the low solubility of polysaccharide–iron complexes in pH 4.5 buffer solution.

#### 3.3.3. Dissolution of Different Polysaccharide–Iron Complex Preparations in Artificial Intestinal Fluid

**Figure 5:**
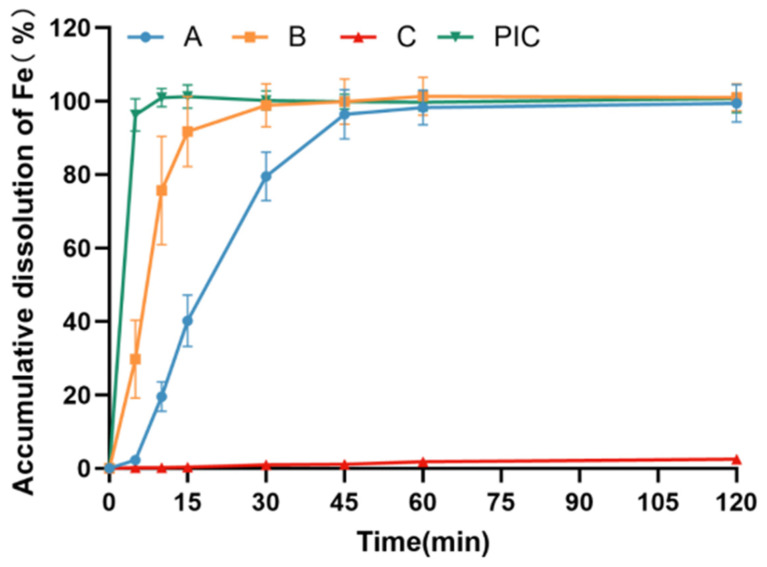
Dissolution curves of different iron preparations in artificial intestinal fluid. Test sample A: coated pellets in imported Niferex capsules, 150 mg; test sample B, powders in capsules, 150 mg; test sample C: ferrous succinate tablets, film coating tablets, 100 mg; PIC: powders, the active ingredients in imported Niferex capsules.

The results illustrated in Figure 5 demonstrate pronounced variations in the dissolution behavior of distinct iron preparation samples in artificial intestinal fluid. Specifically, the dissolution rate of organic iron preparations remained below 5% after 2 h in artificial intestinal fluid, whereas the dissolution rates of the polysaccharide–iron complex preparations and the raw PIC material were notably faster. In fact, the accumulated dissolution rate of iron exceeded 75% within 30 min for both polysaccharide–iron complex preparations, with polysaccharide–iron complex test sample B exhibiting a significantly higher dissolution rate compared to test sample A within the first 30 min (*p* < 0.01). The rapid dissolution characteristics of the active pharmaceutical ingredients (PICs) are apparent, with the granulated test sample A exhibiting a slight reduction in its dissolution rate compared to test sample B over a brief duration.

#### 3.3.4. Dissolution of Different Polysaccharide–Iron Complex Preparations in pH 7.5 Buffer Solution

**Figure 6:**
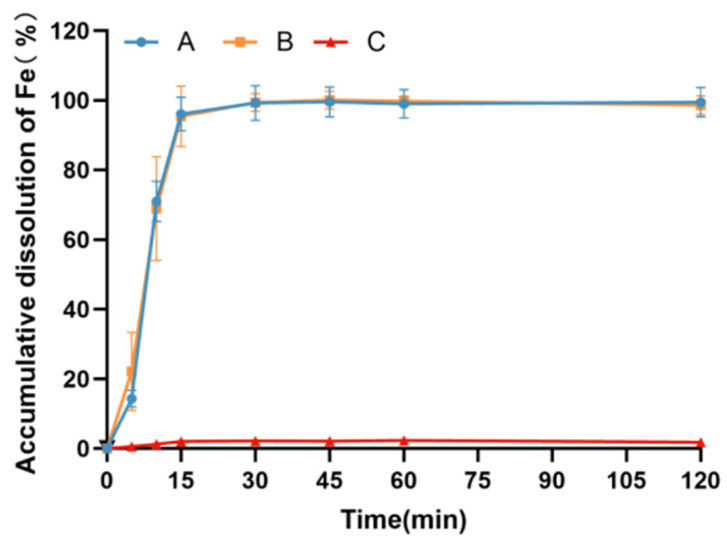
Dissolution curves of different iron preparations in pH 7.5 buffer. Test sample A: coated pellets in imported Niferex capsules, 150 mg; test sample B, powders in capsules, 150 mg; test sample C: ferrous succinate tablets, film coating tablets, 100 mg.

The results presented in Figure 6 reveal notable disparities in the dissolution behavior between the polysaccharide–iron complex preparations and the organic iron preparation in the standard dissolution medium of pH 7.5. Specifically, the dissolution rate of the organic iron preparation remained below 2% after 2 h, whereas the dissolution rate of the polysaccharide–iron complex preparations was notably faster. In fact, the cumulative dissolution rate of iron exceeded 90% within 15 min for the polysaccharide–iron complex preparations. Notably, no significant difference was observed in the dissolution behavior between the two polysaccharide–iron preparations.

In general, compared to the bulk drug PICs, both polysaccharide–iron complexes A and B exhibited a retarding effect on the iron dissolution of PICs in artificial gastric and intestinal fluids. Specifically, Formulation A, produced via pellet processing, showed a more pronounced reduction in dissolution in artificial gastric juice, with its dissolution rate decreasing from 53.39% to 12.98% within 2 h compared to Formulation B. Consequently, employing pellet technology for the preparations may have offered more notable advantages in achieving a slow-release effect in the artificial gastric fluid and a rapid release effect in the artificial intestinal fluid.

The kinetic models of drug release play a crucial role in formulation optimization, clinical applications, and quality control. Fitting drug release kinetic models can elucidate the specific mechanisms by which drugs are released from formulations, such as diffusion, erosion, permeation, or a combination of these mechanisms. By fitting different kinetic models, the primary factors controlling drug release can be identified. Therefore, we selected three kinetic models (the single-exponential model, the Higuchi model, and the Ritger–Peppas model) to fit the data and determine the trends of various parameters during the drug release process, thereby gaining a deeper understanding of the drug release mechanisms. The fitting results indicate that in the artificial gastric fluid, the dissolution behavior of Formulations A and B was primarily governed by the diffusion mechanism, with some contribution from erosion. However, Formulation B exhibited a faster diffusion rate than Formulation A. In the artificial intestinal fluid, the dissolution behavior of both Formulations A and B was predominantly controlled by the erosion mechanism. The high R² values of the single-exponential model suggest that the dissolution processes followed first-order kinetics. However, Formulation A had a slower diffusion rate, which may be attributed to the physicochemical properties of its materials. Table 2 presents the results of in vitro release model fitting for different iron preparations.

#### 3.3.5. Free Iron Content During Dissolution of Different Iron Preparations

**Figure 7:**
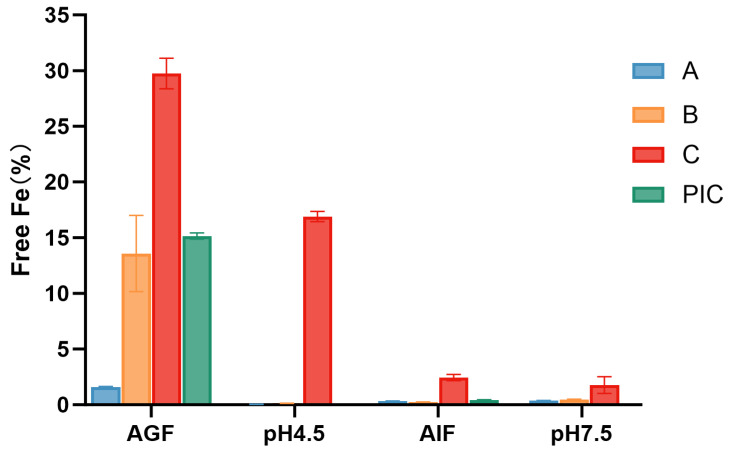
Comparison of free iron contents during dissolution of different iron preparations. Test sample A: coated pellets in imported Niferex capsules, 150 mg; test sample B, powders in capsules, 150 mg; test sample C: ferrous succinate tablet, film coating tablets, 100 mg; PIC: powders, the active ingredients in imported Niferex capsules.

As depicted in Figure 7, the analysis of free iron content during dissolution revealed notable differences between test sample A and polysaccharide–iron complex test sample B in gastric fluid over 2 h. Specifically, in test sample A, the free iron content accounted for 12% of the dissolved amount, representing approximately 1.6% of the total iron content of the preparation. In contrast, polysaccharide–iron complex test sample B exhibited a higher free iron content, constituting 25% of the dissolution amount, or approximately 13.6% of the total iron content. Remarkably, the absolute free iron content in test sample B was 8.5 times greater than that of test sample A.

These results suggested that the utilization of pellet preparation, as seen in test sample A, could significantly reduce the release of free iron by the polysaccharide–iron complexes upon exposure to gastric acid. This reduction in free iron content may help mitigate the potential irritative effects on the gastrointestinal tract, thus enhancing the overall tolerability of the formulation.

### 3.4. In Vivo Pharmacokinetics in IDA Beagle Dogs

**Figure 8:**
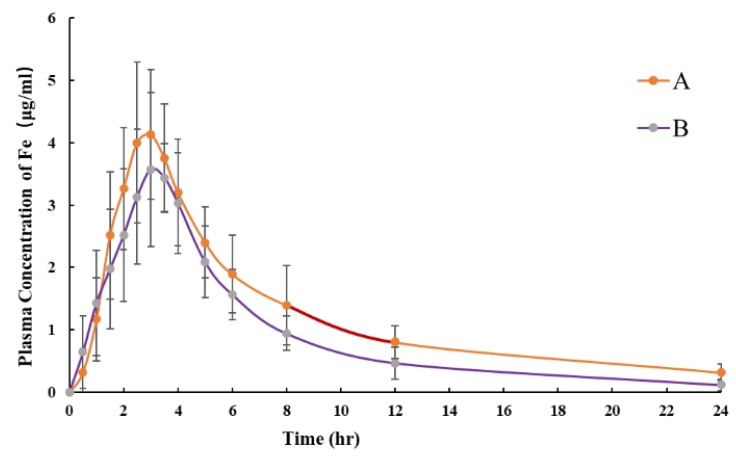
Mean plasma Fe concentrations and standard deviations after single oral doses of different polysaccharide–iron complex capsules were administered to beagle dogs.

After oral administration to IDA beagle dogs, the pharmacokinetic profiles of the polysaccharide–iron complexes were evaluated and compared. The main pharmacokinetic parameters and plasma-concentration profiles are summarized in Table 3 and Figure 8. Although there was no significant difference between sample A and sample B regarding their pharmacokinetic parameters, such as C_max_, T_max_, and t_1/2_ (Table 3), the C_max_ of sample A was much higher and the t_1/2_ was also longer than that of sample B. In addition, the AUC_0-t_ and AUC_0–∞_ of sample A were both significantly higher than those of sample B (*p* < 0.01), and the relative bioavailability of sample B was 78.53% (calculated with AUC_0-t_) and 73.38% (calculated with AUC_0–∞_).

## 4. Discussion

The iron response element/iron regulatory protein (IRE/IRP) system primarily regulates how the intestine absorbs iron. IRP1 and IRP2 respond to changes in cellular iron levels through different mechanisms. They regulate the expression of the iron import transporter DMT1 [21,22,23]. Hepcidin, a peptide hormone produced in the liver, plays a crucial role in regulating iron absorption [24,25]. Under various stimuli, such as erythrocyte dilatation [26] and iron-deficiency anemia, the transcriptional function of iron-regulated proteins is inhibited, leading to tissue hypoxia and decreased serum transferrin saturation [27].

Food digestion is a complex dynamic process involving the transport of food through different regions of the gastrointestinal tract. Once entering the gastrointestinal tract, the iron transport system is decomposed in various compartments (oral cavity, stomach, and small intestine), releasing iron. Gastric acid or gastric juice is detrimental to the digestion of most nutrients, especially acid-sensitive substances, before their adsorption in the intestines. Our findings reveal distinct dissolution behaviors among polysaccharide–iron complexes (test sample A and test sample B), ferrous succinate tablets (test sample C), and PICs in different dissolution media. The dissolution rate of test sample A in acidic conditions was significantly lower compared to that of test sample B. The cumulative iron dissolution rate of test sample A in artificial gastric fluid within 2 h was less than 15%, while for test sample B, it exceeded 50%, with considerable variation between batches. In artificial intestinal fluid, both polysaccharide–iron complex formulations achieved a cumulative iron release rate of over 75% within 30 min. Compared with test sample B, the granular test sample A exhibited a slightly reduced dissolution rate in a short period. In phosphate buffers at pH 4.5 and pH 7.5, no significant differences were observed in the dissolution behavior between test samples A and B. In contrast, the dissolution and release of ferrous succinate tablets (test sample C) were markedly pH-dependent. Although these tablets dissolve faster than the polysaccharide–iron complex preparations at pH 4.5, their dissolution rate was significantly lower than that of the polysaccharide–iron complex preparations at pH 7.5 due to their own isoelectric points and low solubility. The differences in dissolution behavior among various polysaccharide–iron formulations are primarily attributed to two main factors: (1) The chelating ability of polysaccharides with respect to iron: In polysaccharide–iron complexes, iron exists in the form of a β-FeOOH long-chain structure, with the -OH groups of polysaccharides chelating to the iron surfaces to form macromolecules. Compared to Formulation B, the polysaccharides in Formulation A exhibit a stronger chelating ability with respect to iron, which is mainly due to the rich functional groups (such as hydroxyl and carboxyl groups) in their structures and the ability to form more stable spatial structures after chelation with iron. (2) Differences in preparation processes: Pellet preparation technology offers significant advantages in reducing gastric mucosa irritation and improving drug bioavailability. Therefore, polysaccharide–iron complex test sample A was prepared using this technology and coated with a functional film on the pill core exterior. This design resulted in relatively slower iron release under simulated gastric conditions, while test sample A exhibited greater iron release in simulated intestinal fluid compared to simulated gastric conditions. The dual-layer structure of the pellets and polysaccharides endowed the iron molecules with a prolonged release profile. After the outer layer degraded, the exposed sugar chains could adhere to the intestinal wall, increasing the residence time of iron in the intestines and thereby enhancing its bioavailability.

Moreover, free iron can cause oxidative damage to cell membranes through the formation of free radicals and promote thrombus formation, exacerbating necrosis and inflammation, which in turn interferes with ulcer healing. When iron intake exceeds the amount required for sustainability, iron supplements may become toxic. Therefore, we measured the free iron content of different samples during the dissolution process and found that the free iron content of test sample A in gastric fluid over 2 h accounted for 12% of the dissolved amount (approximately 1.6% of the total iron content in test sample A). In contrast, polysaccharide–iron complex test sample B accounted for 25% of the dissolved amount (around 13.6% of the total iron content), with the absolute free iron content being 8.5 times higher than that of test sample A. This discrepancy suggests that the chelating effect of polysaccharide–iron complexes (test sample A) on iron ions may be superior in preventing the release of free iron ions. Compared with traditional PICs, the use of pellet formulation technology significantly attenuates the release of free iron downstream of gastric acid, which may enhance its tolerability and reduce irritation to the gastrointestinal tract. In the simulated intestinal fluid, both test samples A and B exhibited significantly lower free iron contents than test sample C, due to the re-adsorption of iron by polysaccharides at higher pH values.

It has been reported that the chelating capacity of polysaccharides for iron is associated with the molecular weight and distribution, structure, and functional groups of the polysaccharides [28]. Moreover, polysaccharides with higher molecular weights exhibit stronger iron-chelating abilities than those with lower molecular weights [29]. As the molecular weight of polysaccharides in polysaccharide–iron chelates increases, the spatial volume of the polysaccharides expands, forming an iron core within the polysaccharide–iron chelates. The polysaccharides bind to the surface of the iron core through their hydroxyl and carboxyl groups acting as ligands. Test sample A had a significantly higher relative molecular weight than test sample B, indicating its stronger iron-chelating ability. This allowed the formation of a stable β-FeOOH iron core, which is conducive to extending its half-life in vivo. Additionally, the stable spatial structure of test sample A prevents the easy dissociation of iron ions, thereby enhancing its maximum plasma concentration in vivo.

Given the significant differences in the dissolution behavior and free iron contents of various iron preparations, it is necessary to further investigate the behavior of different iron preparations in vivo. Iron-deficiency animal models can simulate real human iron-deficiency status through specific feeding management, for instance, by feeding with iron-deficiency feed or using other methods to prepare iron-deficiency anemia models. In this state, animals’ demand for iron increases, and their absorption, utilization, and metabolism of iron agents also undergo corresponding changes [5,30,31]. Therefore, using an iron-deficient animal model can more accurately reflect the pharmacokinetic characteristics of oral polysaccharide–iron complex agents in the human body under iron-deficiency conditions. Pharmacokinetic analysis revealed that the maximum plasma concentration (C_max_) of test sample A was 4.79 ± 0.72 μg/mL, which was significantly higher than that of test sample B. Moreover, the half-life (t_1/2_) of test sample A in vivo was much longer than that of test sample B, indicating its greater stability in vivo and resistance to rapid degradation by the gastrointestinal tract or metabolic system. Additionally, the AUC_0-t_ and AUC_0-∞_ of test sample B were significantly lower than those of sample A (*p* < 0.01), suggesting a shorter duration in the bloodstream, leading to a decreased total plasma concentration. This may be attributed to its faster digestion or metabolism, reducing the accumulation of plasma. It can be seen that the in vivo behavior of different iron preparations is consistent with their in vitro dissolution behavior. The in vitro solubility and release rate of a drug directly affect its concentration in vivo. Compared with test sample B, test sample A had a lower absorption rate in the simulated intestinal fluid over a short period, and its iron molecules exhibited a prolonged release characteristic. This also indicates that it has a longer half-life and bioavailability in vivo.

## 5. Conclusions

This study thoroughly examined the dissolution behavior, free iron contents, and pharmacokinetics of different iron preparations, particularly polysaccharide–iron complexes (PICs) and ferrous succinate tablets. The polysaccharide–iron complexes (test sample A) showed better acid resistance and controlled release characteristics than other iron supplement formulations. The low dissolution rate in gastric acid and the slow-release behavior in intestinal fluid indicate that it is more tolerable to the gastrointestinal tract and causes less irritation. The microsphere formulation technology in test sample A significantly reduced the release of free iron ions, which likely enhanced its bioavailability and pharmacokinetic profile. In contrast, ferrous succinate tablets (test sample C) displayed noticeable pH-dependent dissolution behavior. They released iron more slowly in intestinal fluid, suggesting possible limitations in both bioavailability and gastrointestinal tolerability. The pharmacokinetic evaluation in an iron-deficient beagle animal model further corroborated the superior performance of test sample A. It exhibited higher bioavailability and prolonged the in vivo retention time, aligning with its in vitro dissolution behavior. These results underscore the potential benefits of microsphere formulation technology in enhancing the efficacy and tolerability of iron supplements. In summary, this study offers important insights into how iron is absorbed and how different iron preparations behave in vivo. The findings suggest that the microsphere formulation of polysaccharide–iron complexes (test sample A) offers significant advantages in terms of controlled release, reduced free iron contents, and enhanced bioavailability. Future research should focus on clinical trials to validate these findings in human subjects and explore the potential of microsphere technology for other therapeutic applications. Further investigation into the long-term effects and patient-specific responses to different iron preparations is also warranted to optimize treatment strategies for iron-deficiency anemia.

## Figures and Tables

**Table 1:** Determination of average molecular weight and distribution of polysaccharide–iron complex preparations and PICs.

	A (10 mg/mL)	B (10 mg/mL)	PICs (10 mg/mL)
Mw	136,764	106,035	131,422
Mn	68,544	57,228	69,031.5
PDI	1.7703	1.7621	1.854

**Table 2:** The results of in vitro release model fitting for different iron formulations.

Dissolution Medium	Formulation	Model	Ordinary Differential Equation (ODE)	Fitting Results	R^2^
Artificial Gastric Fluid	A	Single-Exponential Model	Q = Q_∞_(1 − e ^− kt^)	Q = −2.38 × 10^−11^(1 − e^0.22t^)	0.104
		Higuchi Model	Q = K_H_t^1/2^	Q = 0.249t^0.5^ + 0.231	0.933
		Ritger–Peppas Model	Q/Q_∞_ = kt^n^	Q = 0.23t^0.86^	0.945
	B	Single-Exponential Model	Q = Q_∞_(1 − e ^− kt^)	Q = −7.51 × 10^−4^(1 − e^0.09t^)	0.35
		Higuchi Model	Q = K_H_t^1/2^	Q = 0.77t^0.5^ − 1.25	0.995
		Ritger–Peppas Model	Q/Q_∞_ = kt^n^	Q = 0.28t^1.05^	0.992
	C	Single-Exponential Model	Q = Q_∞_(1 − e ^− kt^)	Q = 41.62(1 − e^−0.01t^)	0.996
		Higuchi Model	Q = K_H_t^1/2^	Q = 0.55t^0.5^ + 2.81	0.935
		Ritger–Peppas Model	Q/Q_∞_ = kt^n^	Q = 1.23t^0.69^	0.986
Artificial Intestinal Fluid	A	Single-Exponential Model	Q = Q_∞_(1 − e ^− kt^)	Q = 108.29(1 − e^−0.03t^)	0.943
		Higuchi Model	Q = K_H_t^1/2^	Q = 1.76t^0.5^ + 20.55	0.599
		Ritger–Peppas Model	Q/Q_∞_ = kt^n^	Q = 10.71t^0.49^	0.828
	B	Single-Exponential Model	Q = Q_∞_(1 − e ^− kt^)	Q = 104.24(1 − e^−0.11t^)	0.913
		Higuchi Model	Q = K_H_t^1/2^	Q = 1.15t^0.5^ + 55.51	0.186
		Ritger–Peppas Model	Q/Q_∞_ = kt^n^	Q = 44.94t^0.19^	0.752

**Table 3:** Main pharmacokinetic parameters in IDA beagle dogs (*n* = 8).

Sample	T_max_	C_max_	t_1/2_	AUC_0–t_	AUC_0–∞_
	h	μg/mL	h	μg·h/mL	μg·h/mL
A	2.94 ± 0.42	4.79 ± 0.72	7.94 ± 2.37	29.66 ± 4.40	33.59 ± 6.81
B	2.88 ± 0.64	4.24 ± 0.92	6.80 ± 0.69	22.31 ± 5.86 **	23.50 ± 4.99 **

**: A statistically significant difference at *p* < 0.01

## Data Availability

The data presented in this study are available upon request from the corresponding author.

## Abbreviations

The following abbreviations are used in this manuscript:

PIC	Polysaccharide–iron complex
IDA	Iron-deficiency anemia
SEM	Scanning electron microscopy
AGF	Artificial gastric fluid
AIF	Artificial intestinal fluid
IDD	Iron-deficient diet
CBC	Complete blood count
SD	Standard deviation
LSD	Least significant difference
AUC	Area under the curve
IRE	Iron response element
IRP	Iron regulatory protein
